# A new genus and species of golden coral (Anthozoa, Octocorallia, Chrysogorgiidae) from the Northwest Atlantic

**DOI:** 10.3897/zookeys.668.12203

**Published:** 2017-04-12

**Authors:** Stephen D. Cairns, Ralf T. S. Cordeiro

**Affiliations:** 1 Department of Invertebrate Zoology, National Museum of Natural History, Smithsonian Institution, PO Box 37012, MRC 163, Washington, DC 20013-7012, USA; 2 Programa de Pós-Graduação em Biologia Animal, Universidade Federal de Pernambuco, Av. Prof. Moraes Rego, 1235, Cidade Universitária, Recife, PE, CEP: 50670-901, Brazil

**Keywords:** *Flagelligorgia gracilis*, unbranched octocoral, biserial polyp arrangement, *Radicipes*, southeastern USA

## Abstract

A new genus and species of unbranched golden coral, *Flagelligorgia gracilis*, is described based on several specimens collected off the southeastern coast of the United States. The genus is provisionally included in the family Chrysogorgiidae, pending molecular confirmation. *Flagelligorgia* morphologically resembles other unbranched chrysogorgiids, such as *Distichogorgia*, *Chalcogorgia*, *Helicogorgia* and *Radicipes*, to which it is compared. The type species is illustrated and its distribution mapped.

## Introduction

Chrysogorgiids form a polyphyletic family of octocorals (Pante et al. 2012), which is distributed in all oceans throughout the world (Watling et al. 2011). Most of its genera are in need of revision, some of which require reallocation into new families (Pante et al. 2012). In the course of doing a revision of the genus *Radicipes* Stearns, 1883 (see Cordeiro et al. in press), an unusual species was encountered that was similar to species in that genus that were already known, but was consistently different from them in several characters. Although no recently collected specimens were available for molecular analysis, sufficient alcohol-preserved specimens were present at the NMNH to describe the new species and the new genus in which it is placed.

## Material and methods

All specimens are preserved in ethanol (70%) and deposited at the National Museum of Natural History. Reference material of all species of *Radicipes* was also examined (Cordeiro et al. in press). The terminology used in the description follows that of Bayer et al. (1983). Preparation of polyps for SEM to reveal sclerites includes short digestion (four seconds) of superficial coenenchyme, rinsing several times in distilled water, and drying at room temperature.

Abbreviations used in the text include: *Alb* – USFWS *Albatross*, L:W – length to width ratio of a sclerite, MCC – Monophyletic Chrysogorgiidae Clade *sensu* Pante et al. (2012), NMNH – National Museum of Natural History, Smithsonian Institution, USNM – United States National Museum (now known as the NMNH, but acronym still used for catalog numbers).

## Taxonomy

### Subclass Octocorallia Haeckel, 1866

#### Order Alcyonacea Lamouroux, 1812

##### Suborder Calcaxonia Grasshoff, 1999

###### Family Chrysogorgiidae

Taxon classificationAnimaliaAlcyonaceaChrysogorgiidae

Verrill, 1883

Chrysogorgidae Verrill 1883: 21.
Chrysogorgiidae : Versluys 1902: 2–4; Bayer 1956: F216; Bayer and Muzik 1976: 67–69 (key to genera); Bayer 1979: 876–878 (key to genera); Cairns 2001: 748–754 (synonymy); Pante and France 2010: 600 (key to genera); Watling et al. 2011: 68–74 (distribution maps of all genera); Pante et al. 2012: 1–12 (phylogenetic and bathymetric analyses).

####### Type genus.

*Chrysogorgia* Duchassaing & Michelotti, 1864.

####### Diagnosis

(after Cairns (2001) and Pante et al. (2012)). Calcaxonians having an unjointed, solid (non-spicular), concentrically layered scleroproteinous axis. The axial layers are usually smooth (not undulated) and thus not longitudinally grooved externally; the axis usually displays metallic or iridescent reflections. The colony may be branched or unbranched (flagelliform), arising from a root-like or discoidal, strongly calcified holdfast. Polyps are contractile but not retractile, arranged in rows (uniserial, biserial or multiserial), but never in whorls. Sclerites predominantly flat, smooth scales, in some species warty rods and spindles.

####### Remarks.

Fourteen genera have been assigned to the Chrysogorgiidae, but based on sequencing of three genes, Pante et al. (2012) have suggested that only six of these genera belong to Chrysogorgiidae *sensu stricto*, which they call the Monophyletic Chrysogorgiidae Clade, or MCC. They imply that the other genera may belong to as many as three other, as yet undescribed, families. The six genera of the MCC are keyed by Pante and France (2010) and their worldwide distributions plotted by Watling et al. (2011). They are further discussed by Pante et al. (2012) in the context of a phylogenetic analysis. *Flagelligorgia* is morphologically most similar to *Radicipes*, which is part of the MCC, but lacking material suitable for molecular analysis, the placement of *Flagelligorgia* in that family can only be a suggestion at this time. In fact, we did attempt to sequence four genes (*COI*, *mtMutS*, *28S* and *18S*) from specimens collected in 1964 with no results.

####### Distribution.

Worldwide, 31–4492 m depths (Pante and Watling 2011: 6).

###### Flagelligorgia gen. n.

Taxon classificationAnimaliaAlcyonaceaChrysogorgiidae

http://zoobank.org/0C142EEB-5AD6-4EE5-A654-F0C5DD669E79

####### Type species.

*Flagelligorgia gracilis*, here designated. Gender: feminine.

####### Diagnosis.

**Figure 1.**
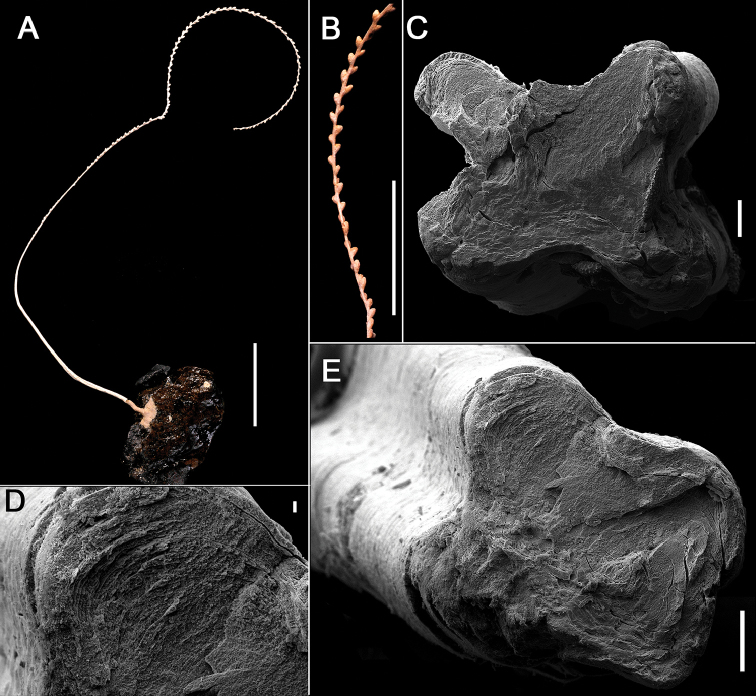
Diagnostic characters of *Flagelligorgia gracilis* sp. n. **A** general view of the holotype (USNM 49503) attached to a rocky substrate **B** detail of the holotype showing the biserial arrangement of polyps **C,E** cross-section of the stem of the holotype showing its four rounded longitudinal cords **D** detail of undulating concentric layers of the stem in cross-section. Scale bars: **A**: 10 mm, **B**: 5 mm, **C, E**: 0.1 mm, **D**: 0.01.

**Figure 2.**
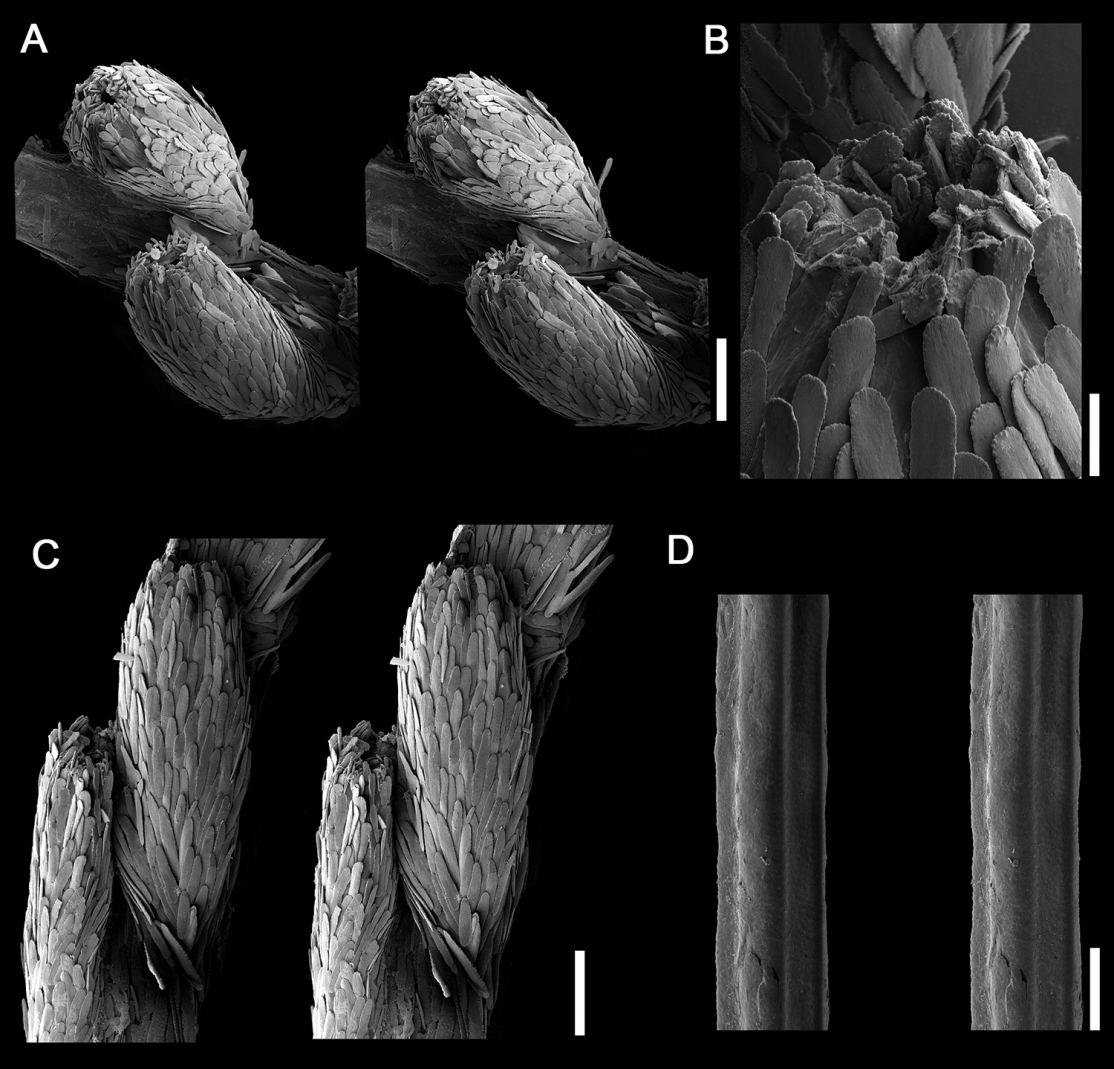
Detailed view of polyps and axis in *Flagelligorgia gracilis* sp. n. (USNM 14458) through Scanning Electron Microscopy. **A** stereo view of polyps **B** oral view of a polyp **C** stereo view of a polyp’s abaxial side **D** stereo view of the axis. Scale bars: **A, C**: 0.2 mm, **B**: 0.04 mm, **D**: 0.01 mm.

Colonies unbranched (flagelliform), loosely coiled, attached by a holdfast; axis composed of undulating concentric layers (Figs 1C–E, 2D). Polyps arranged biserially. Body wall and coenenchymal scales elongate, smooth scales. Polyps lack an operculum.

####### Remarks.

Until 1956 (Bayer 1956) the Chrysogorgiidae had been divided into three subfamilies, one being the Lepidogorgiinae Versluys, 1902, consisting of genera with an unbranched colony and lacking polyp opercula. However, the subfamily classification has been ignored for decades and is not supported by molecular evidence. Nonetheless, it is incumbent to compare *Flagelligorgia* to the four other unbranched chrysogorgiid genera. *Helicogorgia* Bayer, 1981, known only from the east coast of Africa at 66–775 m depth range, is unbranched but has its polyps arranged in a multiserial fashion on one side of the stem, a naked track displayed on the opposite side. Furthermore, its body wall scales are irregular plates and its coenenchymal scales are spindles. For all of these reasons *Helicogorgia* is easily distinguished from *Flagelligorgia*. Based on sequencing of three genes, Pante et al. (2012) indicate that *Helicogorgia* might constitute a family on its own, as sister to the Chrysogorgiidae. *Helicogorgia* was revised by Williams (1992), recognizing four species in the African coast.

*Chalcogorgia* Bayer, 1949, known only from its type locality off northwestern Cuba at 708 m depth, is also unbranched and has biserially arranged polyps, like *Flagelligorgia*, but differs in having eight triangular opercular scales on each polyp, and body wall scales shaped as irregularly shaped platelets. According to Pante et al. (2012: 8), based on “very limited data,” *Chalcogorgia* was suggested to ally with *Helicogorgia*, and thus also outside of the MCC.

*Distichogorgia* Bayer, 1979, known only from the Blake Plateau off Florida at 814 m depth, is unbranched and has biserially arranged polyps, but differs in having unique body wall scales consisting of two fans of longitudinally arranged, blade-like scales, one on each side of the polyp, interspersed with tiny pointed sclerites, similar to its coenenchymals. Pante et al. (2012) did not report sequencing data for this genus, but based on its biserially polyps, they suggested an affinity with *Helicogorgia* and *Chalcogorgia* in a family separate from the Chrysogorgiidae.

Perhaps most similar to *Flagelligorgia* is the genus *Radicipes* Stearns, 1883, consisting of 9–10 species (see Cordeiro et al., in press), which occurs worldwide at depths of 241–3580 m. *Radicipes* is unbranched but differs from *Flagelligorgia* in having uniserially arranged polyps, and a body wall and coenenchymal sclerites that are rod-shaped to compressed rod-shaped. Furthermore, chrysogorgiids (MCC), including *Radicipes*, are characterized by having an axis formed by non-undulated (smooth) concentric layers (Bayer 1956), which is not the case for *Flagelligorgia*. Based on molecular evidence Pante et al. (2012) placed *Radicipes* firmly in the Chrysogorgiidae clade (MCC), as the sister genus to *Chrysogorgia*. Lacking molecular evidence for *Flagelligorgia* (all specimens are quite old), it is not possible at this time to determine if it is allied with *Radicipes* in the Chrysogorgiidae (based on similarity of body wall sclerites) or the other cluster of genera including *Helicogorgia, Chalcogorgia* and *Distichogorgia* (based on polyp arrangement), which may constitute an as yet undescribed family.

####### Distribution.

Off Southeastern United States from North Carolina to Florida, 196–567 m depths.

####### Etymology.

The name is a combination of *flagellum* (Latin for small whip) and *gorgia* (a common octocoral suffix and once the name of the order Gorgonacea, the name derived from the mythical female monster, the Gorgon), in reference to the flagelliform nature of the colony.

###### Flagelligorgia gracilis sp. n.

Taxon classificationAnimaliaAlcyonaceaChrysogorgiidae

http://zoobank.org/579269D2-B012-4C73-A854-E743F7154252

Figs 1 , 2 , 3 , 4

####### Material examined

**(Types).** Holotype: *Alb*-2666, 1 specimen now in two pieces, USNM 49503. Paratypes: *Alb*-2601, 34°39'15"N, 33°30'10"W, 196 m, 1 colony, USNM 16607, 18 October 1885; *Alb*-2602, 34°38'30"N, 75°33'30"W, 227 m, 1 colony, USNM 16821, 18 October 1885; *Alb*-2666, 30°47'30"N, 79°49'W, 494 m, 33 colonies, SEM stubs 2364–2367, USNM 14458, 5 May 1886; *Alb*-2667, 30°53'N, 79°42'30"W, 499 m, 40 colonies, USNM 14457, 5 May 1886; *Gerda*-179, 27°41'N, 79°11'W, 549–567 m, 9 colonies, USNM 57315, 1 July 1963; *Megalopa*, 11.2 km SSE of Carysfort Reef, Florida Keys, 205 m, 9 July 1950, 1 colony, USNM 51956, 9 July 1950; *Pillsbury*-105, 31°00'N, 79°42'W, 388–403 m, 2 colonies, USNM 57316, 27 July 1964; *Pillsbury*-197, 27°59'N, 79°20'W, 567–586 m, 2 colonies, USNM 52913, 11 August 1964.

####### Type locality.

30°47'30"N, 79°49'W (continental slope off Georgia), 494 m depth.

####### Description.

**Figure 3.**
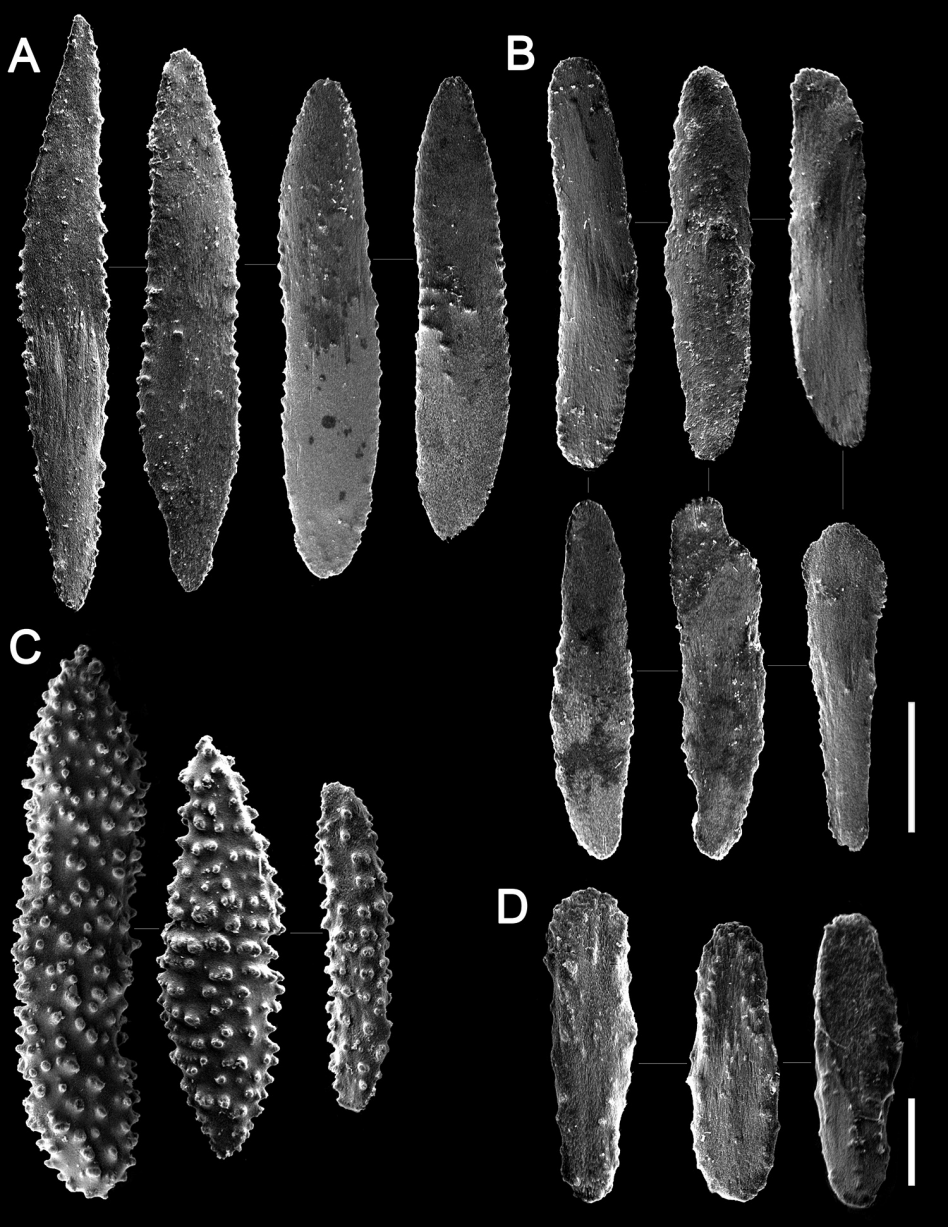
Sclerites of *Flagelligorgia gracilis* sp. n. (USNM 49503, holotype). **A** rods from coenenchyme **B** elongate rods from body wall **C** highly granular scales from first centimeters of the stem **D** tentacular scales. Scale bars: **A–C**: 0.05 mm, **D**: 0.02 mm.

The colony is unbranched (flagelliform) and quite delicate, with an ascending clockwise spiral growth form (Fig. 1A, 2C). One of the largest specimens (the holotype) is 19 cm in length and only 1.1 mm in basal stem diameter. Colonies are attached to hard objects such as the deep-water coral *Lophelia pertusa* (Linnaeus, 1758), rocks, or echinoid tests, having a thin basal encrustation (holdfast) up to 7 mm in diameter; there are no root-like holdfasts. The axis is longitudinally grooved (Fig. 2D), resulting in four rounded longitudinal cords (1C, E), reflecting the underlying undulated layers of scleroprotein (Fig. 1D), most easily seen in stem cross section. The axis is golden, the coenenchymal tissue usually pale brown to a dull yellow, in ethanol. Polyps are lacking from the proximal 45–50 mm of the stem, which is approximately 1/5–1/3 of the colony length, depending on its maturity (Fig. 1A). Polyps occur biserially, on opposite sides of the stem in alternating fashion (Figs 1B, 2A, C), and are relatively closely spaced such that 1.1–1.4 polyps occur per cm length. The polyps are 0.9–1.2 mm in length and are somewhat cigar-shaped (Fig. 2C), being slightly tapered distally; the greatest diameter (0.35–0.39 mm) is usually at mid-length. The body wall is covered with longitudinally oriented, elongate (L:W = 4.5–5.8), imbricating scales (Fig. 3B) that measure 0.17–0.24 mm in length. They are rounded distally, have smooth lateral edges, and are quite thin (e.g., 13–15 µm in thickness). Their outer and inner surfaces are smooth. Toward the end of the polyp are smaller scales associated with the tentacles (Figs 2B, 3D), similar in shape to the body wall scales but only 0.075–0.10 mm in length. Pinnular sclerites are virtually absent. Scales show concentric bands of interference colors in polarized light. There are no sclerites in the axial sheath of coenenchyme. The outer coenenchymal scales (Fig. 3A) are also longitudinally arranged on the stem, elongate (L:W = 4.8–7.9), and imbricate. They have pointed tips and their lateral edges are slightly serrate, each serration up to 5 µm in height; they are also quite thin, and their faces are also smooth. Coenenchymal scales on the first few centimeters of the stem are highly granular (Fig. 3C).

####### Remarks.

As in other unbranched octocorals, such as species of *Radicipes*, some specimens (e.g., from USNM 14458, USNM 51956 and USNM 14457) host commensal ophiuroids. Other octocoral species found at the same stations at which *Flagelligorgia* were collected include: *Plumarella aurea* (Deichmann, 1936), *Plumarella pourtalesii* (Verrill, 1883), *Plumarella dichotoma* Cairns & Bayer, 2004, *Swiftia casta* (Verrill, 1883) and *Callogorgia americana* Cairns & Bayer, 2002.

####### Distribution.

**Figure 4.**
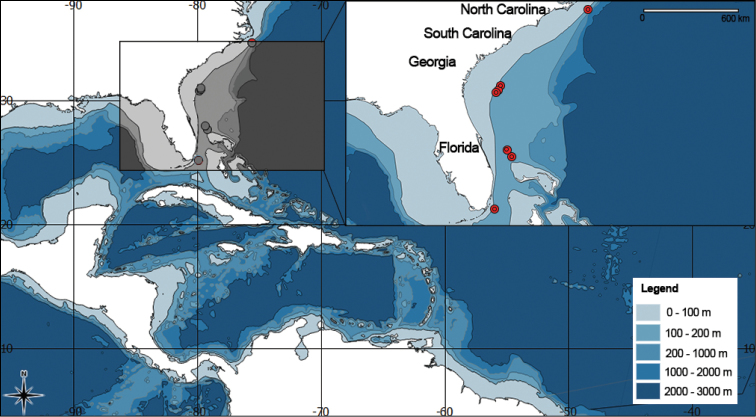
Distribution map of *Flagelligorgia gracilis* sp. n.

Southeastern coast of United States from off Outer Banks, North Carolina to off Carysfort Reef (near Key Largo, Florida) (Fig. 4), 196–567 m depths.

####### Etymology.

Named *gracilis* (Latin for slender, gracile), in allusion to the very slender aspect of the colony.

## Supplementary Material

XML Treatment for Chrysogorgiidae

XML Treatment for Flagelligorgia

XML Treatment for Flagelligorgia gracilis
